# Family burden and happiness in mothers of children with intellectual and developmental disabilities: role of support and quality of life

**DOI:** 10.1192/bjo.2026.11035

**Published:** 2026-04-21

**Authors:** Adile Emel Sardohan Yildirim, Özlem Altindağ Kumaş, Halime Miray Sümer Dodur, Metin Aris, Şenay Delimehmet Dada

**Affiliations:** Department of Special Education, Akdeniz University, Antalya, Turkey; Department of Special Education, Dicle University, Diyarbakir, Turkey; Department of Social Work, Cankiri Karatekin University, Cankiri, Turkey; Department of Special Education, Trabzon University, Trabzon, Turkey

**Keywords:** Intellectual and developmental disabilities, family burden, social support, quality of life, happiness

## Abstract

**Background:**

This study investigates the relationships between family burden, perceived social support, quality of life and happiness among mothers of individuals with intellectual and developmental disabilities (IDDs).

**Aims:**

This study aimed to examine the relationship between family burden and maternal happiness, and to investigate the serial mediating roles of perceived social support and family quality of life in mothers of children with IDDs.

**Method:**

The study sample comprised 250 mothers of children with IDDs. Data were collected using validated instruments: The Multidimensional Scale of Perceived Social Support, a family burden scale, the Beach Center Family Quality of Life Scale and the Oxford Happiness Questionnaire Short Form. Descriptive statistics, correlation analyses and structural equation modelling were conducted with software SPSS 26.0 and JASP 0.16.4.

**Results:**

Perceived social support and family quality of life were positively associated with happiness and negatively associated with caregiving burden; structural equation modelling indicated that their sequential mediation fully explained the link between family burden and happiness.

**Conclusions:**

The findings underscore the critical role of social support and family quality of life in mitigating the negative impact of caregiving burden on maternal happiness.

Intellectual and developmental disabilities (IDDs) refer to a group of conditions that occur during the developmental period, and are characterised by significant limitations in both intellectual functioning and adaptive behaviour.^1^ These limitations manifest themselves in conceptual, social and practical domains, and severely affect individuals’ daily functioning and long-term independence. According to the DSM-5, individuals with IDDs need ongoing support throughout their lives in performing basic life skills and social participation. The global prevalence of intellectual disabilities is estimated to be approximately 2**–**3%, and is even higher in low-income countries because of limited access to health services and inadequate preventive services.^1^

Families of individuals with IDDs, especially primary caregivers, face constant challenges in maintaining daily care routines, accessing adequate services and coping with the emotional and physical burden of caregiving. Research consistently shows that these mothers are at high risk of experiencing stress, anxiety and depression.^2,3^ As children transition into adulthood, difficulties in accessing appropriate services exacerbate family stress.^3^ In addition, caregiving often coincides with the ageing of mothers, creating a dual burden that negatively affects family functioning.

Mothers take on a disproportionate share of caregiving responsibilities and become more vulnerable to negative health outcomes. Studies show that mothers with intellectual disabilities are at higher risk of experiencing health problems such as sleep disorders, musculoskeletal disorders and depression.^4–7^ The decrease in the time that caregivers have for themselves negatively affects both their physical and emotional health, which leads to a decrease in quality of life and an increase in the burden of care.^8^

The biopsychosocial model proposed by Engel^9^ provides a comprehensive theoretical framework for understanding human functioning through the dynamic interaction of biological, psychological and social systems. Within the context of IDDs, this model offers a useful lens to conceptualise how child-related characteristics (biological domain), maternal emotional and cognitive processes (psychological domain), and access to formal and informal support systems (social domain) interact to shape caregiver outcomes. Therefore, caregiving burden and maternal well-being should not be understood as isolated constructs, but rather as outcomes emerging from interconnected systemic influences.

## Social support and caregiver well-being

Social support plays a critical role in the psychological adjustment processes of mothers caring for children with IDDs. Perceived social support refers to the emotional, informational or practical help that individuals perceive they receive from their social environment. Studies show that perceived social support alleviates the negative psychological effects of the care process by increasing coping skills and reducing emotional burden.^10^ Low levels of social support were found to be associated with depressive symptoms, post-traumatic stress and negative parenting behaviours.^11,12^

The protective effect of social support is frequently emphasised in international literature. For example, Lunsky et al^13^ found that social support is a preventive factor for burnout. Studies conducted in different cultural contexts show that strong social networks increase psychological resilience and increase life satisfaction in mothers of children with developmental disabilities.^5,14–16^ A study conducted in South Korea^17^ emphasised that social support reduces the burden of caregiving and promotes personal development, and a study conducted in the USA^11^ found that higher levels of perceived social support were associated with more positive parenting outcomes and reduced family conflict.

In Turkey, because of the limited availability of formal support services, informal support – such as extended family, circle of friends or groups of mothers in similar situations – becomes much more decisive.^18^ Recent studies^19,20^ highlight the importance of awareness-raising education programmes to empower families and reduce the burden of care. When families are informed and supported, they can develop more positive coping strategies and participate more actively in their children’s developmental processes.

## Family quality of life, happiness and care burden

Family quality of life refers to the holistic well-being of family members, which is constituted by their level of satisfaction with various life domains such as health, emotional well-being, financial stability and social relationships.^4,21^ In families with children with IDDs, family quality of life is affected by many variables such as the level of the child’s disability, the socioeconomic status of the family, access to services and the age, education and coping skills of the mothers.^5,18^

As the child’s level of disability increases, mothers’ life satisfaction and psychological well-being have been found to decrease.^1^ It is stated that young mothers adapt to the care process more easily, and an increase in the level of education may differentiate the perception of quality of life depending on the context. In addition, emotional, economic and temporal care stress, defined as ‘family burden’, increases in the absence of adequate formal or informal support, and accordingly, happiness and perception of quality of life decrease.^3^

Happiness, as a core component of subjective well-being, is frequently reported to be lower among mothers caring for individuals with IDDs. The ongoing demands of caregiving, restrictions in personal autonomy, and sustained physical and emotional strain may compromise psychological well-being in this population. In contrast, the presence of emotional support, opportunities for social participation and adequate economic resources have been associated with higher levels of happiness and overall well-being.^13^

The biopsychosocial model proposed by Engel^9^ provides a comprehensive framework for understanding human functioning through the dynamic interaction of biological, psychological and social domains. Applied to the caregiving context, this model suggests that child-related disability characteristics (biological domain), maternal coping processes and emotional regulation (psychological domain), and access to formal and informal support systems (social domain) jointly shape caregiver outcomes. Accordingly, caregiving burden should not be conceptualised solely as an individual psychological experience; rather, it emerges from the interplay of multiple systemic influences. From a biopsychosocial perspective, these domains are inherently interdependent, and caregiving burden may operate both as a contextual stressor and as a mechanism indirectly influencing maternal happiness.

Studies in countries such as Canada, Australia and The Netherlands emphasise that increased family quality-of-life levels are positively associated with children’s developmental outcomes. Caregiver well-being is directly linked to the quality of parenting and the quality of the home environment.^11,22,23^ Therefore, improving family quality of life and reducing the burden of caregiving are critical not only for mothers, but also for children’s long-term developmental outcomes.

## Current study

In the existing literature, these variables have generally been examined independently.^13^ However, studies that address these variables in a holistic manner for primary caregiver mothers are quite limited. A deeper understanding of the interactions between social support, family quality of life and happiness may enable the development of more effective interventions for care processes.^11,21,24^ This study aims to examine the relationships between quality of life, happiness, perceived social support and family burden in mothers of individuals with IDDs from a correlational perspective. The study aims to contribute to the development of social support-based intervention strategies for mothers of individuals with IDDs. In this direction, the following questions were sought to be answered:

- Is there a mediating role of perceived social support between family burden and happiness of individuals with IDDs?
- Is there a mediating role of quality of life between family burden and happiness of individuals with IDDs?
- Is there a serial mediating role of quality of life and perceived social support between family burden and happiness of individuals with IDDs?

## Method

### Participants and procedure

A total of 250 mothers of children with IDDs participated in this study. Participants were recruited through online parent networks and disability support groups, using a convenience sampling approach. Data were collected voluntarily via an online questionnaire. The obtained sample size surpassed the minimum requirements for structural equation modelling, and was deemed statistically sufficient for the analyses conducted. The demographic characteristics of the participants are detailed in Table 1.

**Table 1:** Characteristics of the study group

Characteristic	*n*	%
Number of children		
One child	36	14.4
Two children	70	28.00
Three children	70	28.00
Four or more children	74	29.6
Age		
18–25 years old	3	1.20
26–32 years old	49	19.60
33–40 years old	124	49.60
≥41 years old	74	29.60
Child age		
0–3 years old	5	2
4–6 years old	65	26.8
7–10 years old	65	26.8
11–14 years old	47	18.5
15–17 years old	68	27.4
Education status		
Primary school	59	23.60
Middle school	52	20.80
High school	90	36.00
University	47	18.80
Master’s degree	2	0.8
Employment status		
Not working	127	50.8
Working	123	49.2
Socioeconomic level of the neighbourhood		
Lower socioeconomic	121	48.40
Middle socioeconomic	126	50.40
Upper socioeconomic	3	1.20
Second disability in addition to the child’s intellectual disability		
Emotion and behaviour disorder	11	4.40
Physical disability	52	20.80
Attention-deficit hyperactivity disorder	15	6.00
Speech and language difficulties	20	8.00
Visual impairment	26	10.40
Hearing impairment	33	13.20
Autism spectrum disorder	42	16.80
Chronic diseases	51	20.40

Percentages exceed 100% because some children had more than one additional diagnosis.

### Procedure

The data collection stages were carried out using tools created using Google Forms and accessible via a shareable link. In the informed consent form, families were asked not to share personal information and the purpose of the study was clearly stated. The duration of the questionnaire was set at 25**–**30 min, and mothers were given the right to withdraw if they did not participate in the study or felt uncomfortable after participating. The question ‘Do you want to participate in the study voluntarily?’ on the informed consent form were presented with yes or no options. When determining their participation preferences, participants were asked to check for yes or no boxes. The scales in Google Forms were designed to be accessible during the data collection period and then closed for data entry. As all form items were mandatory, no missing data occurred and no form was considered invalid. Participants were assured that their responses would remain confidential and anonymous and would only be used for research purposes. This study used a convenience sampling approach, as participants were recruited through online parent networks, disability support groups and social media platforms. Because participation was voluntary and depended on internet access and mothers’ willingness to engage, the sample may not fully represent all mothers of children with IDDs in Turkey.

### Ethics statement

The authors assert that all procedures contributing to this work comply with the ethical standards of the relevant national and institutional committees on human experimentation and with the Helsinki Declaration of 1975, as revised in 2013. All procedures involving human participants were approved by the Ethics Committee of Akdeniz University (approval number: 26.11.2024, 24/493).

Ethical approval was obtained before the data collection process. Informed consent was obtained from all participants before they took part in the study. Since the study involved mothers of children with IDDs and did not directly involve children, parental or guardian consent was not required for the children themselves. Participants were informed that their participation was voluntary, that they could withdraw at any time, and that their data would remain anonymous and confidential. Data collection from family for this study was conducted with the utmost consideration for ethical standards.

The study protocol was reviewed and approved by the appropriate educational authorities, ensuring that the research adhered to ethical guidelines and safeguarded the well-being of the participants. Participation in the study was entirely voluntary, and every effort was made to create a comfortable and supportive environment for the participants. Written informed consent was obtained from all participants by providing detailed information about the purpose, process, and possible effects of the study. During the consent process, it was clearly stated that the participants had the right to withdraw from the study at any stage, and that this decision would not lead to any negative consequences. In addition, all participants were assured that their data would be kept confidential and that the research results would be anonymised. This approach ensures that participants’ rights and privacy are protected and guarantees that the research is conducted in accordance with ethical principles.

### Measures

#### Beach Center Family Quality of Life Scale

The Beach Center Family Quality of Life Scale is an instrument devised by the Beach Center on Disability at the University of Kansas, consisting of 25 questions organised into 5 domains;^11^ the validity, reliability and adaptation study conducted by Meral and Cavkaytar^22^ produced the Turkish adaptation of the scale, which consists of five subdimensions: family interaction, parenting, emotional competence, physical/financial/material competence and support for disability. The highest score that can be obtained for the entire 5-point Likert-type scale consisting of a total of 25 items is 125, and the lowest score is 25. High scores indicate a high level of perception of family quality of life and low scores indicate a low level of perception of family quality of life. The internal consistency reliability coefficient of the scale was found to be 0.92.

### The Oxford Happiness Questionnaire Short-Form

The Oxford Happiness Questionnaire was first developed by Argyle et al in 1989, as a 29-item structure. Later, it was revised by Hills and Argyle,^10^ and the Oxford Happiness Questionnaire Short-Form was created. The adaptation, validity and reliability study of the scale into Turkish was conducted by Doğan and Akıncı Çötok.^6^ The scale is a self-report-based, seven-item, five-point Likert-type scale (1 = strongly disagree, 5 = strongly agree) used to determine the subjective happiness levels of individuals. The total score that can be obtained from the scale varies between 7 and 35, and high scores indicate that the happiness level of the individual is high.

For the happiness construct, item parcelling was applied to improve model parsimony in the structural equation model, and two parcels (F1 and F2) were created using a balanced parcelling approach.

### The Multidimensional Scale of Perceived Social Support

The Multidimensional Scale of Perceived Social Support (MPSS) was developed by Zimet et al.^25^ to determine the adequacy of social support perceived by adolescents, and was adapted into Turkish by Eker et al.^7^ The MPSS is a 12-item inventory that subjectively assesses the adequacy of social support received from 3 different sources: family, friends and an expert. The scale is a seven-point Likert-type inventory (scored between 1 and 7) ranging from ‘absolutely no’ to ‘absolutely yes’. The scale consists of three subdimensions: family subscale (includes items 3, 4, 8 and 11), friend subscale (includes items 6, 7, 9 and 12) and expert person subscale (includes items 1, 2, 5 and 10).^25^ The sum of the scores of each subscale (the lowest score is 4 and the highest score is 28) gives the score of the subscale, and the sum of the scores of all subscales (the lowest score is 12 and the highest score is 84) gives the total score of the scale.

### A Family Burden Assessment Scale

A Family Burden Assessment Scale is a 5-point Likert-type measurement tool consisting of 6 sub-factors and 43 items, developed by Yıldırım Sarı, H. and Başbakkal,^26^ to develop a valid and reliable family burden assessment scale for families with IDDs children. The subscales are economic, physical, emotional, social burden, perception of inadequacy and time requirement. The options are scored as ‘1 = never, 2 = rarely, 3 = sometimes, 4 = most of the time/frequently, 5 = always’. High scores indicate that family burden is high. The cut-off point is nine points. The Cronbach’s *α* reliability coefficient of the scale is 0.92.

### Data analysis

Before the main analyses, the data distribution was examined and found to be suitable for parametric analyses. Descriptive statistics, correlation analyses, reliability coefficients (Cronbach’s *α*, McDonald’s *ω* and Guttman’s *λ*), and convergent and discriminant validity analyses were conducted. All preliminary analyses were performed using IBM SPSS Statistics version 26.0 for Windows (IBM Corp., Armonk, New York, USA; https://www.ibm.com/products/spss) and JASP version 0.16.4 for Windows (JASP Team, Amsterdam, The Netherlands; https://jasp-stats.org/).

Structural equation modelling was employed to test the research questions following the two-stage approach proposed by Anderson and Gerbing.^2^ First, the measurement model was evaluated, followed by testing of the structural model. Model fit was assessed with multiple indices, including the standardised root mean square residual (SRMR), root mean square error of approximation (RMSEA), comparative fit index (CFI), normalised fit index (NFI) and incremental fit index (IFI). In line with commonly accepted guidelines, SRMR and RMSEA values below 0.08, and CFI, TLI, NFI and IFI values close to or above 0.90, were considered indicative of acceptable model fit.^27^

## Results

### Preliminary analyses

Table 1 presents the descriptive statistics and bivariate correlations among the study variables. Perceived social support was positively correlated with family quality of life (*r* = 0.88, *p* < 0.001) and happiness (*r* = 0.72, *p* < 0.001), and negatively correlated with family burden (*r* = −0.61, *p* < 0.001). Family quality of life was negatively associated with family burden (*r* = −0.68, *p* < 0.001) and positively associated with happiness (*r* = 0.76, *p* < 0.001). In addition, happiness was negatively correlated with family burden (*r* = −0.63, *p* < 0.001).

Preliminary analyses indicated that the data met the assumptions for parametric analyses. Skewness and kurtosis values were within acceptable ranges, internal consistency coefficients exceeded 0.80 for all scales and no evidence of multicollinearity was observed. Descriptive statistics and reliability coefficients for all study variables are presented in Table 2.

**Table 2:** Relationships between variables and descriptive statistics

Variables	1	2	3	4
1. Perceived social support	–			
2. Quality of life	0.88**	–		
3. Family burden	−0.61**	−0.68**	–	
4. Happiness	0.72**	0.76**	−0.63**	–
Arithmetic mean	44.24	66.13	162.26	17.97
s.d.	22.38	29.82	37.26	4.62
Skewness	0.06	−0.14	−0.91	0.32
Kurtosis	−1.45	−1.46	0.24	−0.23
Cronbach’s *α*	0.93	0.98	0.91	0.80

**p < 0.001.

### Structural equation modelling

First, the measurement model including 4 latent and 16 observed variables was tested and demonstrated good model fit: *χ* ^2^(98, *N* = 250) = 508.00, *χ* ^2^/d.f. = 3.18, RMSEA = 0.065, SRMR = 0.058, TLI = 0.932, CFI = 0.944, NFI = 0.919 and IFI = 0.945. All indicators loaded significantly on their respective latent constructs, and reliability coefficients were high (*α* ≥ 0.80).

Following confirmation of the measurement model, the structural model was examined. In the partial mediation model, the direct path from family burden to happiness was not statistically significant (*B* = 0.10, *p* > 0.05). Although the NFI was slightly below the conventional 0.90 threshold (0.899), overall model fit was acceptable, consistent with recommendations for complex models in social science research.^27^

Consequently, the direct path was removed and the full mediation model was tested. The full mediation model showed good fit (*χ* ^2^(99, *N* = 250) = 525.20, *χ* ^2^/d.f. = 3.23, RMSEA = 0.061, SRMR = 0.061, TLI = 0.930, CFI = 0.942, NFI = 0.918, IFI = 0.943), and all structural paths were statistically significant.

**Fig. 1:**
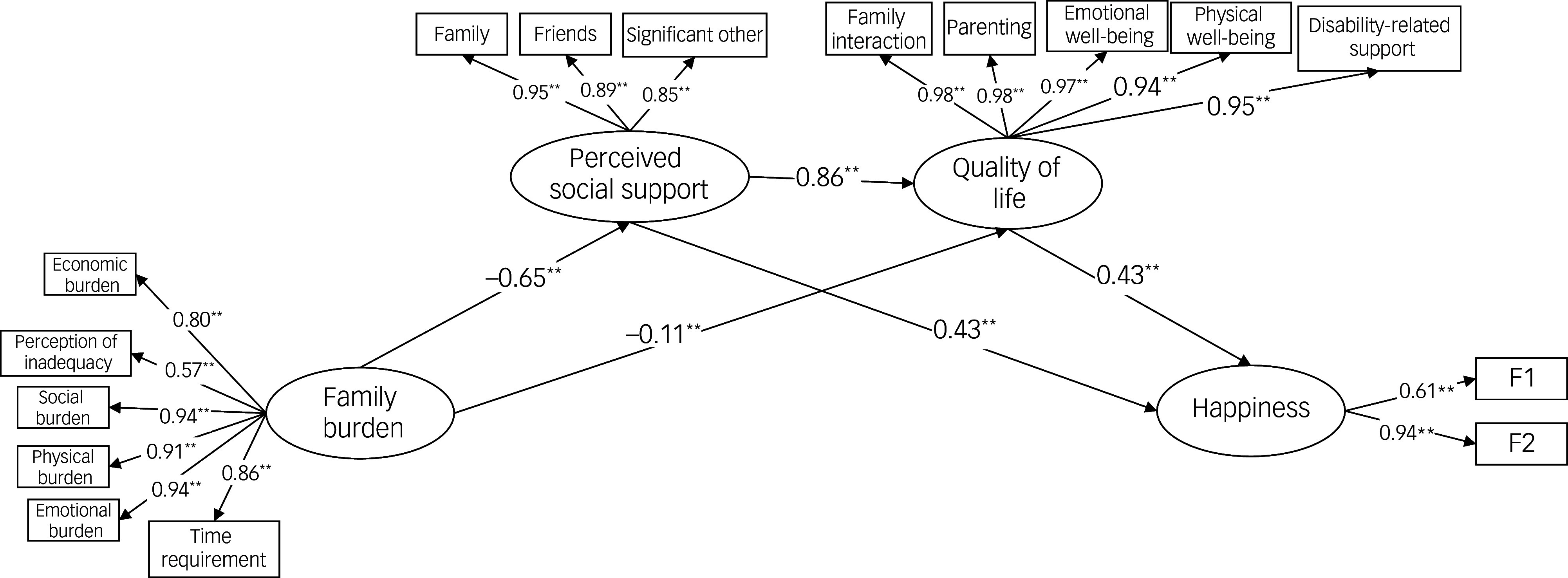
Structural equation model of the serial mediation analysis. F1 and F2 indicate the two item parcels used to represent the happiness construct. **p < 0.01; F1–F2 parcel of happiness.

Bootstrap analyses with 5000 resamples indicated that the indirect effect of family burden on happiness through perceived social support (*B* = −0.82, 95% bias-corrected and accelerated confidence interval (BCa CI) −1.18 to −0.55) and through family quality of life (*B* = −0.68, 95% BCa CI −0.91 to −0.47) were significant. Moreover, the sequential mediation of perceived social support and family quality of life fully accounted for the relationship between family burden and happiness (*B* = −0.059, 95% BCa CI −0.113 to −0.085). The final model is presented in Fig. 1.

## Discussion

This study adds to the growing body of research examining the well-being of mothers caring for children with IDDs, by integrating four key constructs – family burden, social support, family quality of life and happiness – into a single serial mediation model. This approach provides a clearer understanding of the mechanisms underlying caregivers’ well-being. By examining the relationships between quality of life, happiness, perceived social support and family burden in mothers of individuals with IDDs, this study provides a holistic perspective to understand the psychological well-being of caregiving mothers. Although the findings are consistent with both national and international literature, they also provide some unique contributions. Given the cross-sectional design, the study examines associations between variables rather than establishing causal relationships; therefore, expressions referring to ‘effects’ should be interpreted as statistical associations rather than causal pathways.

First, strong and positive relationships were found between perceived social support and both quality of life and happiness. This finding supports previous studies emphasising the importance of social support as a protective factor.^4,9,28^ In particular, Lunsky et al^13^ found that families with multiple care responsibilities experience high levels of stress and burnout, which increases the need for support. In the Turkish context, informal support networks (e.g. extended family, circle of friends, mothers with similar experiences) are known to be more decisive because of the limited availability of formal support services.^19,26^ In this context, in our study, it was observed that high perceived social support increased life satisfaction and decreased care burden. This reveals that not only the quantitative presence of social support, but also how it is perceived by the individual, is critical.

The finding of a negative relationship between perceived social support and family burden in the study points to the alleviating effect of supportive social relationships on care burden. This finding overlaps with the results of Oh and Lee,^17^ who emphasised the role of social support in reducing care burden in their study conducted in the South Korean context. Similarly, Kay et al’s^20^ study in the USA shows that higher levels of perceived social support are associated with more positive parenting outcomes and reduced family conflict. These findings suggest that social support plays a universal protective role in different cultural contexts, and that it is even more decisive in countries with limited service infrastructure, such as Turkey.

Another important finding of the study was that there were significant negative correlations between family burden and quality of life and happiness. This result coincides with the work of Dimitrova-Radojichikj,^5^ who found that the perception of quality of life and happiness decreases when family burden is high. In addition, it has been emphasised in various studies that care burden is disproportionately distributed, especially for women, and that this is associated with negative health outcomes.^7,9,19^ This study shows that the negative effects on mothers’ health, psychological well-being and social lives are felt more intensely as the family burden increases. In fact, as Weiss et al^29^ stated, the decrease in the time that the caregiver has to spare for themselves leads to both physical and emotional burnout, which negatively affects life satisfaction.

Structural equation modelling findings show that the relationship between family burden and happiness is not direct, but indirect, through perceived social support and quality of life. This suggests that caregiver mothers’ happiness levels are not only related to the burden they are under, but also to how they cope with this burden and what kind of support systems they have access to. Although Engel^9^ stated that chronic stress, loss of autonomy and physical fatigue decrease levels of happiness, other studies have shown that social participation, emotional support and financial stability are associated with higher happiness and well-being.^13,28,30^ Similarly, in this study, social support and quality of life stand out as determinants of mothers’ happiness. It should also be acknowledged that constructs such as family quality of life and perceived social support may partially overlap conceptually, as supportive family environments inherently contribute to both perceived social support and overall quality-of-life assessments. Future studies could employ alternative measurement models to further distinguish these dimensions.

Another noteworthy finding of the study is that quality of life is positively related to happiness and negatively related to family burden. This result shows that family quality of life is a multidimensional structure that affects not only individual satisfaction, but also the well-being of all family members. As Staunton et al^31^ stated, family quality of life is not limited to health status or material conditions; it also includes elements such as social relationships, emotional competence and parenting roles. According to the findings of Alnahdi and Schwab,^1^ an increase in the child’s level of disability decreases the mother’s life satisfaction. Similarly, in this study, it was observed that quality-of-life scores decreased as care burden increased.

The impact of quality of life on mothers’ happiness is a frequently emphasised issue in the literature. For example, Kay et al^20^ and Qian et al^32^ found that family quality of life is directly related to the quality of parenting and the quality of the home environment. Similarly, Jansen et al^19^ reported that long-term developmental outcomes of children in families with high quality of life are also positively affected. In this context, improving quality of life is considered a critical goal not only for parental well-being, but also for children’s development.

Another important contribution of the study is the ability to fully explain the effect of family burden on happiness through the sequential mediation of social support and quality of life. This result supports the assumption that factors interact with each other and form a complex network of relationships based on the biopsychosocial model. In particular, it should be noted that stressors such as care burden are shaped by environmental and structural resources (social support, service access), as well as individual variables (coping strategies, self-efficacy).^13,23,29^ These findings may also be associated with sociodemographic characteristics such as age, education level and employment status. As stated by Borilli et al,^4^ parental education, access to services and coping skills affect quality of life. In the current study, a significant portion of the sample was university graduates and came from a middle socioeconomic level, and that may have contributed to the high perception of social support and relatively positive quality-of-life scores.

When evaluated in the cultural context, it is seen that the care processes of individuals with IDDs in Turkey are generally based on women, public supports are insufficient and caregiving mothers have difficulty in benefiting from support systems. As emphasised by researchers such as Green^8^ and Weiss et al,^29^ this care burden should be defined as a burden that has not only physical, but also emotional and social dimensions. The results of this study reveal that it is possible to alleviate the effects of this burden in the Turkish context through social support.

### Limitations and suggestions

This study has several limitations that should be considered when interpreting the findings. First, its cross-sectional design precludes causal inferences among study variables. Although significant associations were identified, longitudinal studies are required to clarify the directionality of the relationships between family burdens, perceived social support, quality of life and happiness. Second, all data were collected using self-report measures, which may be subject to social desirability and response biases, particularly for subjective constructs such as happiness and quality of life. In addition, data collection was conducted online, and only mothers with internet access were able to participate, which may limit the representativeness of the sample. Finally, the sample primarily consisted of mothers from middle socioeconomic backgrounds. Therefore, the findings may not be generalisable to low-income, rural or less educated populations, whose access to support systems and caregiving experiences may differ.

Despite these limitations, our findings have important implications for research and practice. Strengthening informal and formal social support systems may improve the quality of life and well-being of mothers who care for individuals with IDDs. Psychoeducation and emotional support–based interventions developed in collaboration with public institutions and civil society organisations may help reduce caregiving burden and enhance coping skills, particularly when designed with cultural sensitivity.

Future research should include more diverse and representative samples and consider involving other family members, such as fathers, siblings or grandparents, to better understand role-sharing and burden distribution within families. Longitudinal and intervention-based studies are also valuable for examining causal mechanisms and evaluating the effectiveness of support-oriented programmes.

In conclusion, this study has made significant contributions to the literature by addressing the factors determining the psychological well-being of mothers caring for children with IDDs, in a multidimensional way. The main findings of the study show that social support and quality of life have direct and indirect effects on mothers’ well-being, and family burden plays a role as a critical variable in these relationships.

This study reveals that mothers with IDDs experience higher happiness levels when they perceive strong social support and maintain a high family quality of life. Conversely, caregiving burden negatively affects their well-being. A key finding is that caregiving burden does not directly reduce mothers’ happiness; instead, its impact is fully mediated by perceived social support and family quality of life. This highlights the pivotal buffering role of supportive social networks and a positive family environment in mitigating strain. For individuals with intellectual disabilities, the study underscores the indirect benefits of empowering caregivers through social support systems, as enhanced maternal well-being is associated with better caregiving outcomes and family functioning. For the research community, the study contributes a comprehensive model demonstrating the sequential mediation effect, offering empirical evidence to guide future intervention designs focused on improving both caregiver and care recipient outcomes within disability contexts.

This study makes two main contributions to the literature. First, by examining these variables within a serial mediation model, it presents a comprehensive framework that captures the complex mechanisms linking caregiving burden and well-being. Second, the results provide practical implications for the design of family-centred interventions. Strengthening informal and formal support systems, enhancing families’ access to psychosocial resources and developing culturally sensitive support programmes may substantially improve the well-being of caregiving mothers, and indirectly support positive developmental outcomes in children with IDDs.

## Data Availability

Researcher’s who wish to obtain additional data or conduct further analysis may contact the relevant authors to request supplementary information or materials. The corresponding author, A.E.S.Y., is willing to collaborate and provide the necessary information to support the results presented in the article.
